# Royal Free Hospital

**Published:** 1909-02-20

**Authors:** 

**Affiliations:** President. Sandwich, Chairman of the Committee


### EDITOR'S LETTER-BOX.

### ROYAL FREE HOSPITAL.

*To the Editor of* The Hospital.

Sir,—We venture to ask your assistance in making known that, by permission of the Lord Mayor, the annual meeting of the Royal Free Hospital, Gray's Inn Road, will be held at the Mansion House on March 10 next, at 3 p.m., when the claims of the hospital for increased public support will be advocated. The demands made upon the resources of the hospital by the sick poor increase year by year. During the past year there were 2,213 in-patients and 36,358 out-patients, casualties, and maternity cases. The total attendances of patient during the year were 101,241. As is indicated by its title, admission to the hospital is entirely free. No subscribers' letters are required, and no charge whatever is made to the patients. The upkeep last year cost £17,632, while the reliable income did not exceed one-third of that sum. It is only by strenuous exertions that the committee have managed hitherto to pay their way, and they are extremely anxious to maintain the hospital without getting into debt. It has been necessary during the past few years to expend large additional sums upon structural and other improvements in order to maintain the efficiency of the hospital. In these circumstances the committee earnestly ask the support of the charitable public at the Mansion House meeting, so as to enable them to carry on the beneficent work of the hospital. Subscriptions will be thankfully received by Lloyds Bank at any of its branches, by the Treasurers, and also by the Secretary at the hospital.

We are, yours faithfully,
Helena, President.
Sandwich, Chairman of the Committee.

February 5.

